# Dental prosthetic treatment needs of inpatients with schizophrenia in Taiwan: a cross-sectional study

**DOI:** 10.1186/1472-6831-13-8

**Published:** 2013-01-19

**Authors:** Kuan-Yu Chu, Nan-Ping Yang, Pesus Chou, Lin-Yang Chi, Hsien-Jane Chiu

**Affiliations:** 1 Department of Dentistry, Tao-Yuan General Hospital, DOH, Tao-Yuan, Taiwan; 2 Institute of Public Health & Community Medicine Research Center, National Yang-Ming University, Taipei, Taiwan; 3 Department of Geriatrics, Tao-Yuan General Hospital, DOH, Tao-Yuan, Taiwan; 4 Department of Dentistry, National Yang-Ming University, Taipei, Taiwan; 5 Department of Psychiatry, Jianan Mental Hospital, DOH, Tainan, Taiwan

**Keywords:** Fixed prosthesis, Removable prosthesis, Treatment needs, Schizophrenia

## Abstract

**Background:**

The need to obtain information on the dental prosthetic treatment needs (DPTNs) of inpatients with schizophrenia is unrecognized. This study aims to assess the DPTNs of this population and investigate the association between these needs and related factors.

**Methods:**

The results of an oral health survey involving 1,103 schizophrenic adult inpatients in a long-term care institution in Taiwan were used. Chi-square tests and multiple logistic analyses were used to measure the independent effects of the characteristics of each subject on their DPTNs.

**Results:**

Of the subjects, 805 (73.0%) were men and 298 (27.0%) were women. The mean age was 50.8 years. A total of 414 (37.5%) required fixed prosthesis, whereas 700 (63.5%) needed removable prosthesis. Multivariate analyses show that fixed prosthesis is associated with age only after adjusting for other potential independent variables. Older subjects who had a lower educational attainment or a longer length of stay required removable prosthesis.

**Conclusions:**

The findings of this study show that the DPTNs of schizophrenic inpatients are not being met. Therefore, a special approach to the dental prosthetic treatment of these patients should be developed.

## Background

Dental prosthesis is very important for patients with post-endodontic therapy and who are partially or completely edentulous because it improves their chewing ability, digestion, aesthetics, and quality of life. The need to obtain information on the dental prosthetic treatment needs (DPTNs) of handicapped individuals in general [1-3] and of inpatients with psychiatric disorders in particular has been gaining recognition [4,5].

Schizophrenia is a severe psychiatric disorder that has a significantly negative effect on the self-care ability of patients, which leads to poor oral hygiene as well as high DPTNs [6,7]. Previous studies found high levels of dental treatment needs in schizophrenic populations [8].

The factors that affect dental prosthetic treatments have not been appropriately recognized. Researchers have reported that gender has a significant influence on the need for a fixed or removable prosthesis [9]. Studies have also indicated that age, educational level, and length of stay (LOS) in institutions are important factors that determine dental treatment needs [8,10,11]. Schizophrenic patients that require dental treatment often come from poor families with limited access to dental care. Moreover, the dental care provided to patients who stay in long-term-care institutions for a considerable amount of time is insufficient [6,12]. Furthermore, some schizophrenia medications, such as the typical antipsychotic drugs, may have side effects that can lead to increased DPTNs [13,14].

Professional clinical examinations are reportedly more effective than self-assessment or questionnaire-based screenings in assessing individual dental treatment needs [3,15], particularly if done with the aid of radiographs [16,17]. The planning of preventive oral health improvement strategies based on DPTNs as well as on related factors associated with a population is important. However, few studies have been conducted on the DPTNs of hospitalized patients with schizophrenia based on clinical oral examinations via periapical radiography. Schizophrenic inpatients probably belong to a disadvantaged minority and thus receive inadequate dental care. The aim of this study is to obtain descriptive epidemiological information on the DPTNs of hospitalized schizophrenic patients and to identify the association between DPTNs and several potentially related factors that affect these patients.

## Methods

### Participants

This study involved a sample hospital that offers long-term medical care for chronic psychotics. The hospital is located in eastern Taiwan but receives psychiatric patients from all over the country. As of mid-2006, the hospital had admitted 2,330 patients. In recruiting subjects for the study, all hospital patients with schizophrenia as the main psychiatric diagnosis (ICD-9 code: 295) were selected to undergo a standardized DPTN assessment in the hospital’s Department of Dentistry. A total of 1,468 schizophrenic patients were involved in the study. Of these, 1,103 (response rate: 75.1%) participated and completed the DPTN evaluation. Ethical clearance was obtained from the Ethical Committee of Yuli Hospital, Department of Health (DOH), Taiwan, and all participants gave informed consent.

### DPTN assessment

DPTN assessment was performed under standard conditions using a dental chair with adequate light, proper dental instruments, and periapical radiographs, as needed. A trained dentist performed all oral examinations with the help of an experienced assistant, who recorded the DPTN data as well as information on independent variables.

The DPTN items used were consistent with those recommended by the World Health Organization, [18] which included (1) fixed prosthesis (FP); and (2) removable prosthesis (RP), such as a removable partial denture (RPD; those crossing the midline were defined as bilateral RPD) and complete dentures.

The principle behind dental prosthetic treatment is the restoration or replacement of all lost teeth, excluding third molars. An FP is generally preferred because the reported survival rate of patients with FPs is higher than that of patients with removable RPDs [19,20]. Thus, FPs are the first choice for replacing missing teeth in partially edentulous patients.

An FP consists of a single crown and a bridge. The single crown is a cemented veneer restoration that replaces a tooth suffering from deep decay or undergoing post-endodontic therapy [21]. The selection of the abutment tooth for the bridge follows Ante’s Law, which states that the root surface area of the abutment teeth should equal or surpass that of the pontics [22]. An FP with cantilever extension units usually has higher failure rates than conventional designs [23]; thus, a bridge with an end abutments was used in this study. Dental implants were excluded from the treatment plans because of financial limitations.

On the other hand, an RP is recommended for patients whose remaining teeth lack the strength to support a fixed bridge. RPs are classified into partial dentures and complete dentures. RPDs that do not cross the midline are classified as 0.5 units.

### Independent variables

The independent variables included age, educational attainment, marital status, grade of disability, use of antipsychotic medication, and LOS. Age was classified into four strata: 20–44, 45–54, 55–64, and over 65 years old. Educational attainment was divided into two categories: 6 years and below, and above 6 years. Marital status was classified into three categories: single, married, and separated/divorced/widowed. Disability was divided into three grades: mild or moderate (moderate), severe, and profound**.** These grades are in accordance with the disability certificate issued by the Taiwan Ministry of Interior. The use of antipsychotic medications was stratified into two categories based on records obtained for six months prior to the study period [24]. Antipsychotic medications comprised first-generation antipsychotics (FGAs, e.g. chlorpromazine, clopenthixol, flupenthixol HCl, haloperidol, loxapine, sulpiride, and thioridazine) and non-FGA medications. Non-FGAs included second-generation antipsychotics (SGAs, e.g. clozpaine, olanzapine, risperidone, and quetiapine) and third-generation antipsychotics (TGAs, e.g. aripiprazole). The length of stay (LOS) cut-off point, which refers to the number of years since admission to the hospital, was 10 years [13].

### Data collection and analysis

Data collection was conducted from January to December 2006. The DPTN items were then prepared and summarized. The data files were then designed using MS Access and analyzed using SPSS software version 15.0. Proportions, group means, and standard deviations (SDs) were calculated. Univariate analysis using the chi-square test compared the differences between the independent variables and the DPTNs. Statistical significance was set at the *P* < 0.05 level.

Multiple logistic regressions were used to estimate the association between the independent variables and DPTNs of the participants. The independent variables were included in the multivariable logistic models, with a cut-off of *P* < 0.05 from the univariate analyses. The odds ratios (OR) of the independent variables (as compared to the reference category), the significance (*P*-value), and 95% confidence intervals (95% CI) were estimated from the multiple logistic regression models. Areas under a receiver-operating characteristic curve (AUC) were also calculated to determine the predicted probability of the logistic models.

## Results

### Descriptive statistics

Of the 1,103 schizophrenic inpatients evaluated for DPTNs, 805 (73.0%) were men and 298 (27.0%) were women. The mean age was 50.8 years (SD = 10.8), and the average LOS in the hospital was 8.4 years (SD = 5.7). A total of 55 (5.0%) inpatients were found to be edentulous.

Of the subjects, 414 (37.5%) required FP, with 1,854 units and a mean unit of 1.7 (SD = 2.89) per person. On the other hand, 700 (63.5%) patients required RP, whereas 645 (58.5%) required RPD, including 28 (2.5%) unilateral, 240 (21.8%) bilateral, 26 (2.4%) unilateral combined with bilateral, and 351 (31.8%) two bilateral arches. Moreover, 145 (13.1%) patients required complete dentures, with 93 (8.4%) requiring a single arch and 52 (4.7%) requiring two arches. Table 1 shows the numbers and percentages of DPTNs of the subjects who require FP or RP.

**Table 1:** Number and percentage (within parentheses) of dental prosthetic treatment needs among inpatients with schizophrenia in Taiwan

** Variables**	**Total**	**Fixed prosthesis**	***P *value**	**Removable prosthesis**	***P *value**
**Total**	1103 (100)	414 (37.5)		700 (63.5)	
**Sex**					
Men	805 (73.0)	302 (37.5)	1.000	518 (64.3)	0.325
Women	298 (27.0)	112 (37.6)		182 (61.1)	
**Age (yrs)**					
20-44	301 (27.3)	142 (47.2)	<0.001	127 (42.2)	<0.001
45-54	425 (38.5)	183 (43.1)		272 (64.0)	
55-64	257 (23.3)	67 (26.1)		191 (74.3)	
65+	120 (10.9)	22 (18.3)		110 (91.7)	
**Education (yrs)**					
>6	468 (42.4)	193 (41.2)	0.032	250 (53.4)	<0.001
≤6	635 (57.6)	221 (34.8)		450 (70.9)	
**Marriage**					
Single	848 (76.9)	320 (37.7)	0.657	538 (63.4)	1.000
Married	115 (10.4)	39 (33.9)		73 (63.5)	
Separated	140 (12.7)	55 (39.3)		89 (63.6)	
**Disability**					
Moderate	237 (21.5)	99 (41.8)	0.250	131 (55.3)	<0.001
Severe	744 (67.5)	274 (36.8)		474 (63.7)	
Profound	122 (11.1)	41 (33.6)		95 (77.9)	
**Low income**					
No	240 (21.8)	91 (37.9)	0.940	137 (57.1)	0.023
Yes	863 (78.2)	323 (37.4)		563 (65.2)	
**FGA using**					
No	692 (62.7)	272 (39.3)	0.123	424 (61.3)	0.053
Yes	411 (37.3)	142 (34.5)		276 (67.2)	
**LOS (yrs)**					
≤10	517 (46.9)	215 (41.6)	0.011	272 (52.6)	<0.001
>10	586 (53.1)	199 (34.0)		428 (73.0)	

Abbreviations: *FGA*: first generation antipsychotic; *LOS*: length of stay.

### Univariate analysis

The chi-square test shows that age (*P* < 0.001), educational attainment (*P* = 0.032), and LOS (*P* = 0.011) were associated with the need for FP treatment. In other findings, age (*P* < 0.001), educational attainment (*P* < 0.001), disability (*P* < 0.001), low income (*P* = 0.023), use of FGAs in borderline cases (*P* = 0.053), and LOS (*P* < 0.001) determined the need for RP treatments.

### Multivariate logistic analysis

Based on the multivariate logistic analysis results (Table 2), age was negatively associated with FP. The analysis also reveals that the need for RP was associated with age, educational attainment, and LOS.

**Table 2:** Multivariate analyses of factors associated with dental prosthetic treatment needs among inpatients with schizophrenia in Taiwan

**Variables**	**Fixed prosthesis **^**a**^				**Removable prosthesis RP **^**b**^			
	**B**	**OR**	**P**	**95.0% CI for OR**	**B**	**OR**	**P**	**95.0% CI for OR**
**Age (yrs)**								
20-44		1 (r)				1 (r)		
45-54	−0.16	0.85	0.297	0.63-1.16	0.72	2.06	<0.001	1.50-2.83
55-64	−0.93	0.40	<0.001	0.27-0.59	1.04	2.82	<0.001	1.91-4.18
65+	−1.37	0.25	<0.001	0.15-0.44	2.28	9.75	<0.001	4.78-19.87
**Education (yrs)**								
>6						1 (r)		
≤6					0.40	1.49	0.004	1.13-1.95
**LOS (yrs)**								
≤10						1 (r)		
>10					0.36	1.43	0.016	1.07-1.91
**AUC**			0.62				0.71	

Abbreviations: *LOS*: length of stay; *AUC*: area under curve; *r*: reference.

Variables entered: ^a^ age, education, *LOS*; ^b^ age, education, grade of disability, low income, use of FGA, LOS.

## Discussion

This study investigated the DPTNs and several potentially related factors that affect schizophrenic inpatients using a large sample from a specific psychiatric hospital in Taiwan. The study obtained not only descriptive information on DPTNs, but also indicated that age, education, and LOS are associated with DPTNs in some of the subjects. These findings may help oral health policy decision-makers in allocating adequate resources and in developing effective strategies to meet the DPTNs of this special needs population.

FP was associated with age only after adjusting for other independent variables. Older subjects who had lower educational attainment or had longer LOS required RP. These findings are only partly consistent with those of previous studies on the oral health of inpatients with psychiatric disorders. However, these data sets do complement one another [5,25-28].

We estimated the average budget of prosthetic treatment needs for schizophrenic in-patients. Based on the fee collection policies of Yuli Hospital, DOH, a base-metal crown treatment costs NT$4,000; unilateral RPD, NT$18,000; bilateral RPD, NT$24,000; and a complete denture per arch, NT$27,000. Hence, the average budget for the prosthetic treatment needs of an inpatient is calculated as follows: [1,854 × NT$4,000 + (28+26) × NT$18,000 + (240 + 26 + 351 × 2) × NT$24,000 + (93 + 52×2) × NT$27,000] ÷ 1,103 subjects = NT$33,490. In other words, the cost of providing the prosthetic treatment needs of an average schizophrenic inpatient in Taiwan is approximately US$1,158 (USD).

In terms of human resources, as of July 2010, the ratio of the general population to dentists in Taiwan was 1,995: 1. The availability of only one full-time dentist for 2,300 inpatients with severe psychiatric disorders in this hospital is clearly insufficient.

The acceptance of dental prosthetic treatment for missing teeth would seem reasonable. However, studies proposed a compromise that involves a shortened dental arch. In this treatment, the restoration of four occluding pairs of premolars can provide sufficient occlusal stability and masticatory function [29,30].

Discussion on extended issues such as costs of various prosthetic treatments is beyond the scope of this study, since it entails the value discount, different prices of prosthetic treatments between urban and rural areas, and those between fixed and removable prosthesis of different materials. Furthermore, some limitations of this study should be noted. First, the sample may not be representative of all schizophrenic inpatients in Taiwan. For practical reasons, only a selected number of inpatients were examined. Given that the survey design focused only on schizophrenic patients with DPTNs in a public psychiatric hospital, patients with very limited life expectancy and those not hospitalized were excluded. Second, despite our best efforts, a number of practical difficulties regarding DPTNs were encountered during the assessment. In addition, a large number of schizophrenic patients were unaccounted for during the process. Third, we acknowledge that the evidence supporting this assumption is limited and that further research on the development of an effective preventive program for the unmet DPTNs of schizophrenic inpatients is urgently required.

## Conclusions

The considerable unmet treatment needs of hospitalized schizophrenic patients in Taiwan were observed. Age, educational attainment, and LOS are the three most significant factors associated with the need for RP treatment. The results reveal the need for the development of a prompt oral health care delivery service model for patients with special needs.

## Competing interests

The authors declare that they have no competing interests.

## Authors' contributions

KYC carried out the DPTN assessment, participated in the sequence alignment and drafted the manuscript. NPY participated in the design of the study and performed the statistical analysis. PC and LYC conceived of the study, and participated in its design and coordination and helped to draft the manuscript. HJC participated in the sequence alignment. All authors read and approved the final manuscript.

## Pre-publication history

The pre-publication history for this paper can be accessed here:

http://www.biomedcentral.com/1472-6831/13/8/prepub

## References

[B1] Whyman RA Treasure ET Brown RH MacFadyen EE The oral health of long-term residents of a hospital for the intellectually handicapped and psychiatrically ill N Z Dent J 1995 91 404 49 56 7675347

[B2] Lester V Ashley FP Gibbons DE The relationship between socio-dental indices of handicap, felt need for dental treatment and dental state in a group of frail and functionally dependent older adults Community Dent Oral Epidemiol 1998 26 3 155 159 10.1111/j.1600-0528.1998.tb01943.x 9669592

[B3] Hennequin M Faulks D Roux D Accuracy of estimation of dental treatment need in special care patients J Dent 2000 28 2 131 136 10.1016/S0300-5712(99)00052-4 10666971

[B4] Velasco E Bullon P Periodontal status and treatment needs among Spanish hospitalized psychiatric patients Spec Care Dentist 1999 19 6 254 258 10.1111/j.1754-4505.1999.tb01394.x 11833430

[B5] Kumar M Chandu GN Shafiulla MD Oral health status and treatment needs in institutionalized psychiatric patients: one year descriptive cross sectional study Indian J Dent Res 2006 17 4 171 177 10.4103/0970-9290.29868 17217213

[B6] Friedlander AH Marder SR The psychopathology, medical management and dental implications of schizophrenia J Am Dent Assoc 2002 133 5 603 610 12036166 10.14219/jada.archive.2002.0236

[B7] Yaltirik M Kocaelli H Yargic I Yaltirik M Kocaelli H Yargic I Schizophrenia and dental management: review of the literature Quintessence Int 2004 35 4 317 320 15119719

[B8] Barnes GP Allen EH Parker WA Lyon TC Armentrout W Cole JS Dental treatment needs among hospitalized adult mental patients Spec Care Dentist 1988 8 4 173 177 10.1111/j.1754-4505.1988.tb00726.x 2978775

[B9] Al-Ghannam NA Al-Shammery AR Wyne AH Khan NB Al-Ghannam NA Al-Shammery AR Wyne AH Khan NB Prosthetic dental treatment needs in Eastern [corrected] Saudi Arabia.[Erratum appears in Saudi Med J. 2002 Nov;23(11):1427] Saudi Med J 2002 23 8 975 980 12235473

[B10] Angelillo IF Nobile CGA Pavia M Fazio P Puca M Amati A Dental health and treatment needs in institutionalized psychiatric patients in Italy Community Dent Oral Epidemiol 1995 23 6 360 364 10.1111/j.1600-0528.1995.tb00263.x 8681519

[B11] Chu K-Y Yang N-P Chou P Chiu H-J Chi L-Y Factors associated with dental caries among institutionalized residents with schizophrenia in Taiwan: a cross-sectional study BMC Publ Health 2010 10 1 482 10.1186/1471-2458-10-482 PMC2931468 20707911

[B12] Kilbourne AM Horvitz-Lennon M Post EP McCarthy JF Cruz M Welsh D Blow FC Oral Health in Veterans Affairs Patients Diagnosed with Serious Mental Illness J Public Health Dent 2007 67 1 42 48 10.1111/j.1752-7325.2007.00007.x 17436978

[B13] Thomas A Lavrentzou E Karouzos C Kontis C Factors which influence the oral condition of chronic schizophrenia patients Spec Care Dentist 1996 16 2 84 86 10.1111/j.1754-4505.1996.tb00839.x 9084341

[B14] Szabadi E Tavernor S Hypo- and Hypersalivation Induced by Psychoactive Drugs: Incidence Mechanisms and Therapeutic Implications CNS Drugs 1999 11 449 466 10.2165/00023210-199911060-00004

[B15] Robinson PG Nadanovsky P Sheiham A Can Questionnaires Replace Clinical Surveys to Assess Dental Treatment Needs of Adults? J Public Health Dent 1998 58 3 250 253 10.1111/j.1752-7325.1998.tb03002.x 10101703

[B16] Becker T Levin L Shochat T Einy S Becker T Levin L Shochat T Einy S How much does the DMFT index underestimate the need for restorative care? J Dent Educ 2007 71 5 677 681 17493976

[B17] Heyes G Robinson PG Heyes G Robinson PG Pilot study to assess the validity of the single assessment process as a screening tool for dental treatment needs in older people Gerodontology 2008 25 3 142 146 10.1111/j.1741-2358.2008.00215.x 18341577

[B18] WHO Oral Health Survey: Basic Methods 1997 4 Geneva

[B19] Glantz P-OJ Nilner K Jendresen MD Sundberg H Quality of fixed prosthodontics after 15 years Acta Odontol Scand 1993 51 4 247 252 10.3109/00016359309040574 8237309

[B20] Vermeulen AHBM Keltjens HMAM van't Hof MA Kayser AF Ten-year evaluation of removable partial dentures: Survival rates based on retreatment, not wearing and replacement J Prosthet Dent 1996 76 3 267 272 10.1016/S0022-3913(96)90170-5 8887799

[B21] Shillingburg HT Jr Hobo S Whitsett L Fundamentals of Fixed Prosthodontics: Diagnosis and Treatment Planning. vol. 1 1981 2 Chicago: Quintessence Publishing Co 13 53

[B22] Ante IH The fundermental principles of abutments Mich State Dent Soc Bul 1926 8

[B23] Karlsson S Failures and length of service in fixed prosthodontics after long-term function. A longitudinal clinical study Swed Dent J 1989 13 5 185 192 2683178

[B24] Jansen ENH Clozapine in the treatment of tremor in Parkinson's disease Acta Neurologica Scandinavica 1994 89 4 262 265 10.1111/j.1600-0447.1994.tb01511.x 8042443

[B25] Kenkre AM Spadigam AE Oral health and treatment needs in institutionalized psychiatric patients in India Indian J Dent Res 2000 11 1 5 11 11307250

[B26] Ramon T Grinshpoon A Zusman SP Weizman A Oral health and treatment needs of institutionalized chronic psychiatric patients in Israel Eur Psychiatry 2003 18 3 101 105 10.1016/S0924-9338(03)00023-3 12763294

[B27] Tang WK Sun FCS Ungvari GS O'Donnell D Oral Health of Psychiatric In-Patients in Hong Kong Int J Soc Psychiatry 2004 50 2 186 191 10.1177/0020764004043134 15293435

[B28] Zusman SP Ponizovsky AM Dekel D Masarwa A-e-S Ramon T Natapov L Grinshpoon A An assessment of the dental health of chronic institutionalized patients with psychiatric disease in Israel Spec Care Dentist 2010 30 1 18 22 10.1111/j.1754-4505.2009.00118.x 20051070

[B29] Witter DJ van Palenstein Helderman WH Creugers NHJ Käyser AF The shortened dental arch concept and its implications for oral health care Community Dent Oral Epidemiol 1999 27 4 249 258 10403084 10.1111/j.1600-0528.1998.tb02018.x

[B30] Kanno T Carlsson GE A review of the shortened dental arch concept focusing on the work by the Käyser/Nijmegen group J Oral Rehabil 2006 33 11 850 862 10.1111/j.1365-2842.2006.01625.x 17002745

